# Better Functional Recovery After Single-Level Compared With Two-Level Posterolateral Lumbar Fusion

**DOI:** 10.7759/cureus.23010

**Published:** 2022-03-09

**Authors:** Scott D Daffner, Joshua T Bunch, Douglas C Burton, R. Alden Milam IV, Daniel K Park, K Brandon Strenge, Peter G Whang, Howard S An, Branko Kopjar

**Affiliations:** 1 Department of Orthopedics, West Virginia University School of Medicine, Morgantown, USA; 2 Department of Orthopedic Surgery and Sports Medicine, University of Kansas Medical Center, Kansas City, USA; 3 Orthopedic Surgery, OrthoCarolina Spine Center, Charlotte, USA; 4 Orthopedic Surgery, Michigan Orthopedic Surgeons, Southfield, USA; 5 Spine Surgery, The Orthopaedic Institute of Western Kentucky, Paducah, USA; 6 Department of Orthopedics and Rehabilitation, Yale School of Medicine, New Haven, USA; 7 Department of Orthopedic Surgery, Rush Presbyterian Hospital, Chicago, USA; 8 Department of Health Services, University of Washington, Seattle, USA

**Keywords:** two-level, patient satisfaction, quality of life, outcomes, oswestry disability index (odi), novel bone graft substitute, degenerative, bone graft materials, instrumented posterolateral fusion (plf), single-level

## Abstract

Background

Multiple studies describe the outcomes of patients undergoing single-level and multilevel posterolateral lumbar fusion (PLF). However, a comparison of outcomes between single-level and two-level PLF is lacking. The aim of this prospective cohort study was to compare outcomes between single-level and two-level instrumented PLF.

Methods

A total of 42 patients were enrolled at nine US centers between October 2015 and June 2017. Data included radiologic outcomes, visual analog scale (VAS) Back and Leg Pain, disability per the Oswestry Disability Index (ODI), and health-related quality of life (QoL) per 36-Item Short Form Survey version 2.0 (SF-36v2) at six weeks and three, six, 12, and 24 months.

Results

Twelve-month and 24-month follow-ups were completed by 38 (90.5%) and 32 (76.2%) subjects, respectively. The average age was 67 years, and 54.8% were female. Twenty-six received single-level PLF, and 16 received two-level PLF. In the single-level group, there was one reoperation, two postoperative infections, and one dural tear. In the two-level group, there was one postoperative infection. The surgeon computed tomography (CT)-based evaluation of fusion rate was 67.6% (25/37) at 12-month follow-up and 94.1% (32/34) at 24-month follow-up. The third-party evaluation of fusion rate was 52.8% (19/36) at six months, 81.1% (30/37) at 12 months, and 86.5% (32/37) at 24 months. There was a tendency toward a higher fusion rate in single-level compared with two-level PLF. The ODI, SF-36v2 Mental Component Score (MCS), and VAS Back Pain and Leg Pain outcomes improved by the first follow-up visit in both the single-level and two-level groups. Improvement in the ODI was 5.86 (95% confidence interval (CI): 0.03-11.69) points greater in the single-level group compared with the two-level group.

Conclusions

Compared with the two-level PLF subjects, single-level PLF subjects had better functional outcomes and reported higher satisfaction with the outcome of surgery but showed similar fusion, pain, and generic health-related quality of life outcomes. Both single-level and two-level PLF subjects demonstrated high fusion rates in association with improvements in pain, functional, and quality of life outcomes, as well as high satisfaction levels.

## Introduction

Posterolateral lumbar fusion (PLF) is a common surgical procedure for numerous spinal conditions including degenerative disk disease, spondylolisthesis, and scoliosis. PLF may be performed as a standalone fusion procedure or in combination with an interbody fusion construct. In PLF, the fusion is achieved by facilitating bony bridging between the facet joints and/or across the transverse processes of the adjacent spinal segments.

Multiple studies and systematic reviews have described the outcomes of patients undergoing PLF [1-8]. Furthermore, several studies have reported the outcomes associated with the use of multilevel PLF procedures [9,10]. However, a comparison of outcomes between single-level and two-level PLF is lacking in published literature. The purpose of this analysis was to compare outcomes between single-level and two-level instrumented PLF.

## Materials and methods

Patient population

Patients undergoing instrumented PLF who were enrolled in a prospective multicenter study at nine sites in the USA (clinicaltrials.gov NCT02225444) were included in this analysis. The IRB approval numbers are as follows: 00000482, 1408414269, 00003411, 20152227, 2015-8059 (15-059), 20152227, 1606018020, 20152227, and SLUHN 2016-21. The aim of the study was to evaluate the effectiveness and safety of OSTEOAMP® (Bioventus LLC, Durham, NC, USA) in patients requiring single-level or two-level adjacent instrumented posterolateral fusion of the lumbar or lumbosacral spine. Key inclusion criteria were as follows: i) a diagnosis of degenerative disk disease, degenerative spondylolisthesis (up to grade 1 by Meyerding classification), and/or mild degenerative scoliosis (up to 25 degrees curvature); ii) requiring a PLF of up to two adjacent levels from L1 to S1, and iii) having a preoperative Oswestry Disability Index (ODI) score of more than 30. Patients who received other bone graft substitutes (e.g., bone morphogenetic protein (BMP), stem cells, allograft bone, or autogenous bone) as part of a prior PLF were excluded, as were patients who required interbody fusion. All patients received PLF at one or two spinal levels utilizing local autologous bone augmented with the OSTEOAMP bone graft substitute.

The planned sample size was 120 patients. A total of 56 patients were screened and 42 patients were enrolled between October 2015 and June 2017, at which time enrollment was halted due to slow patient recruitment. We used data from these 42 patients and performed a secondary analysis to compare outcomes between single and two-level fusions (level of evidence: II, prospective cohort study).

The study protocol was approved by the Institutional Review Boards (IRBs) from all nine investigational sites. Prior to participation in the clinical study, each patient provided written informed consent.

Surgical technique

PLF was performed per standard of care at the participating institutions. All patients received pedicle screw and rod instrumentation without the insertion of interbody fusion devices. The OSTEOAMP granules were rehydrated with bone marrow aspirate (BMA) and combined with local autograft bone obtained from the decompression. At least two-thirds of the total graft volume used was to consist of OSTEOAMP. A minimum of 10 cc was planned to be administered per side per level (i.e., at a minimum of 20 cc per level). The bone graft was placed on the decorticated surface of the transverse processes and along the pars interarticularis; facet joint fusion was not required by the protocol but was performed at the treating surgeon’s discretion.

Data collection and quality assurance

Baseline data, including demographic and medical history information, were collected from each patient. At the six-week and three-, six-, 12-, and 24-month in-clinic follow-up visits, data on radiologic outcomes, back and leg pain, disability, and health-related quality of life (QoL) were collected. An outside contract research organization (CRO) verified the integrity, completeness, and authenticity of the data with 100% source data verification performed during on-site visits to the investigational sites.

Anteroposterior (AP) and lateral radiographs were taken at baseline and each follow-up visit. Flexion-extension radiographs were obtained at all follow-up visits except for the six-week and three-month visits. Computed tomography (CT) scans with 1 mm contiguous axial slices without a bone filter and without intravenous (IV) contrast were obtained at the 12-month visit. Window and level settings were optimized for trabecular bone detail, and both coronal and sagittal reconstructions were performed. The CTs were repeated at the 24-month follow-up visit for patients who did not show bilateral fusion on their 12-month CTs.

Endpoints

Fusion was determined by clinical and radiographic assessments of radiographs and CTs by the treating physicians and by a central laboratory (Medical Metrics Inc., Houston, TX, USA). The criteria for radiographic success for this analysis was bridging bone between the transverse processes or facet joints of each treated level as assessed with AP and lateral X-ray as well as CT (if applicable). Fusion was deemed successful at the spinal level if left and/or right posterolateral bridging bone was present and posterolateral cracking was absent on the evaluated side, criteria similar to that published by Christensen et al. [11]. Additionally, the central laboratory determination of fusion success required less than five degrees of angular motion and 3 mm or less of translation. Leg pain and back pain were assessed using a 10-cm visual analog scale (VAS). Functional and disability outcomes were measured using the Oswestry Disability Index (ODI) [12]. QoL outcomes were measured using the 36-Item Short Form Survey version 2.0 (SF-36v2) Physical and Mental Component Scores (PCS and MCS) [13]. Patients were asked about return to activities and satisfaction with surgery at each follow-up visit. Treatment complications were also assessed at each follow-up visit.

Statistical methods

Outcomes were compared between patients who received either single-level or two-level instrumented PLF. Fusion outcomes were compared using Fisher’s exact test. Changes in clinical outcomes were analyzed using the Mixed Model for Repeated Measures (MMRM) analysis, using factors for follow-up visits (six weeks and three, six, 12, and 24 months) and the number of operated spinal levels (one or two levels). All analyses were adjusted for the baseline values of the endpoint. Two patients withdrew from the study prior to the three-month follow-up and were excluded from these analyses. Prior to MMRM analysis, any missing 24-month follow-up values were imputed by carrying forward 12-month follow-up values. Per the study protocol, successful fusion at the 12-month follow-up was carried over to the 24-month follow-up. The analysis had 75% a priori statistical power to detect a difference in improvement in ODI of 15 on a scale from 0 to 100 under the assumed standard deviation of 18. Statistical analyses were performed by an independent statistician. All analyses were performed using SAS for PC version 9.4 (SAS Institute Inc., Cary, NC, USA).

## Results

The 12-month and 24-month follow-up visits were completed by 38 (90.5%) and 32 (76.2%) patients, respectively. The average age was 67 years, and 54.8% were female (Table 1).

**Table 1:** Demographic and Surgical Information *P < 0.05

Demographics	Single level (N = 26)	Two levels (N = 16)
Age (years)	66.3 (SD = 10.9)	68.2 (SD: 9.45)
Sex (female)	13 (50%)	10 (62.5%)
Surgical procedure		
Laminectomy	24 (92.3%)	15 (93.8%)
Foraminotomy	9 (34.6%)	4 (25%)
Approach		
Standard open	25 (96.2%)	15 (93.8%)
Mini-open	1 (3.9%)	1 (6.3%)
Total levels	26	32
L3/L4	0	8
L4/L5	23	16
L5/S1	3	8
Length of hospital stay (days)	2.96 (SD = 1.40)	3.25 (SD = 2.96)
Operative time (minutes)*	207 (SD = 56.9)	272.6 (SD = 89.54)
Blood loss (cc)	323.1 (SD = 298.7)	547.5 (SD = 294.7)
Total graft volume used (cc)*	26.2 (SD = 9.95)	46.8 (SD = 16.95)
Total graft volume used per level (cc)	26.2 (SD = 9.95)	23.4 (SD = 8.47)

Twenty-six (61.9%) patients received a single-level PLF, and 16 (38.1%) received a two-level PLF. The majority of patients (92.9%) underwent laminectomy at one or more levels.

There were no differences between patients receiving the single-level or two-level PLF in terms of age, sex, surgical procedure, spinal levels, length of hospital stay, or blood loss. The duration of surgery was longer in the two-level group compared with the single-level group. As would be expected, the total graft volume was larger in the two-level group; however, when this was standardized per level, there were no differences between the groups (Table 1).

The overall fusion rate based on operating surgeons’ evaluations was 67.6% (25/37) at the 12-month follow-up and 94.1% (32/34) at the 24-month follow-up. There was no significant difference in fusion rates between single-level and two-level procedures (Table 2). The fusion rate based on independent central laboratory measurements was 52.8% (19/36) at six months, 81.1% (30/37) at 12 months, and 86.5% (32/37) at 24 months. The difference between the assessments by the investigators and the central laboratory in the number of evaluations available at 24 months is because surgeons' evaluations were not obtained for the six-month follow-up. There was a trend toward a higher fusion rate in single-level PLFs compared with two-level PLFs (Table 2).

**Table 2:** Fusion Outcomes by Follow-Up Time and Number of Spinal Levels *Per protocol, surgeon evaluations of fusion status were not performed at the six-month follow-up visit.

	Six months	12 months	24 months
Surgeon*			
Single level		16/22 (72.7%)	20/21 (95.2%)
Two levels		9/15 (60%)	12/13 (92.3%)
P-value		0.4879	1.000
Total		25/37 (67.6%)	32/34 (94.1%)
Central laboratory			
Single level	11/22 (50%)	19/22 (86.4%)	20/22 (90.9%)
Two levels	8/14 (57.1%)	11/15 (73.3%)	12/15 (80%)
P-value	0.7419	0.4081	0.3773
Total	19/36 (52.8%)	30/37 (81.1%)	32/37 (86.5%)

Table 3 summarizes patient-reported outcomes.

**Table 3:** Improvement in Functional and Quality of Life Outcomes by Follow-Up Visit and Number of Levels * P < 0.05 for baseline differences between single-level and two-level instrumented PLF N1: number of subjects in the single-level group; N2: number of subjects in the two-level group Numbers are least-square means. Numbers in parentheses are 95% confidence intervals.

			Improvement over baseline				
		Baseline	Six weeks	Three months	Six months	12 months	24 months
		N_1_ = 25; N_2_ = 15	N_1_ = 25; N_2_ = 14	N_1_ = 25; N_2_ = 15	N_1_ = 23; N_2_ = 15	N_1_ = 23; N_2_ = 15	N_1_ = 23; N_2_ = 15
ODI	Single level	52.2 (43.9, 60.4)	26.3 (18.6, 34.0)	31.3 (23.6, 39.0)	32.5 (24.5, 40.5)	35.1 (27.1, 43.1)	33.6 (25.6, 41.6)
	Two levels	51.2 (43.3, 59.1)	19.8 (9.6, 30.1)	25.9 (16.0, 35.8)	30.3 (20.4, 40.2)	27.4 (17.5, 37.3)	26.0 (16.1, 35.9)
SF-36v2 PCS	Single level	32.5 (29.9, 35.1)*	10.2 (6.4, 13.9)	13.5 (9.8, 17.2)	15.7 (11.8, 19.5)	16.1 (12.3, 20.0)	15.4 (11.5, 19.3)
	Two levels	27.9 (25.3, 30.5)	8.8 (3.9, 13.8)	11.8 (7.0, 16.6)	14.1 (9.3, 18.9)	14.1 (9.3, 18.9)	12.7 (7.9, 17.5)
SF-36v2 MCS	Single level	41.5 (35.8, 47.2)	5.8 (1.6, 9.9)	7.4 (3.4, 11.5)	5.5 (1.3, 9.8)	7.5 (3.2, 11.7)	7.9 (3.7, 12.2)
	Two levels	45.0 (38.6, 51.5)	8.5 (3.1, 13.9)	8.4 (3.2, 13.7)	7.2 (2.0, 12.5)	6.6 (1.4, 11.8)	5.6 (0.3, 10.8)
VAS Leg Pain	Single level	6.7 (5.6, 7.9)	4.8 (3.8, 5.8)	4.3 (3.3, 5.3)	5.2 (4.2, 6.2)	4.5 (3.4, 5.5)	4.8 (3.8, 5.8)
	Two levels	6.5 (4.6, 8.3)	4.6 (3.2, 6.0)	5.5 (4.2, 6.8)	5.7 (4.4, 7.0)	5.0 (3.7, 6.3)	4.9 (3.6, 6.2)
VAS Back Pain	Single level	6.5 (5.7, 7.4)	4.5 (3.4, 5.5)	4.2 (3.1, 5.2)	4.8 (3.7, 5.9)	4.8 (3.7, 5.9)	5.0 (3.9, 6.1)
	Two levels	6.4 (5.3, 7.5)	4.8 (3.5, 6.1)	4.5 (3.3, 5.8)	5.3 (4.0, 6.6)	5.0 (3.8, 6.3)	4.7 (3.4, 5.9)

There were no preoperative differences between the groups in terms of ODI, SF-36v2 MCS, and VAS Back Pain and VAS Leg Pain values. Preoperative SF-36v2 PCS values were lower among two-level patients compared with single-level patients. All patient outcomes improved by the first follow-up visit at six weeks in both the single-level and two-level groups. There were no significant improvements noted beyond six weeks in either group.

**Figure 1:**
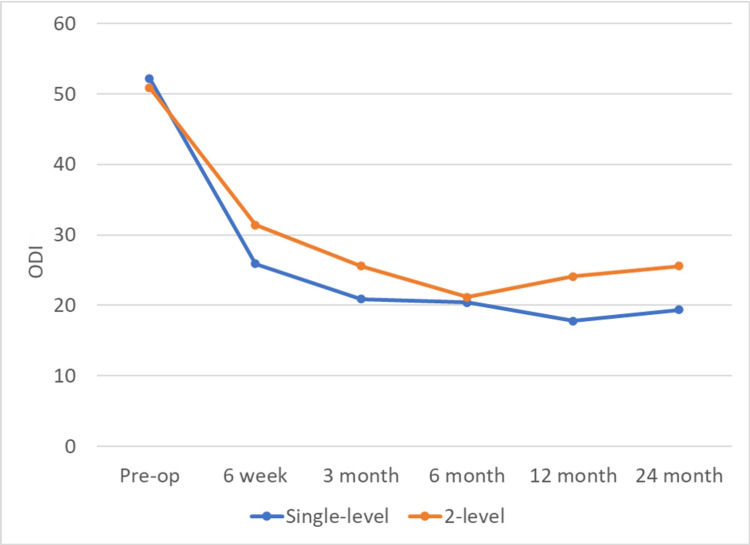
Oswestry Disability Index (ODI) in Single-Level and Two-Level PLF PLF: posterolateral fusion

Overall, improvement in the ODI was greater in the single-level group compared with the two-level group. The average difference in improvement across all follow-up times between the single-level group and the two-level group was 5.86 (95% confidence interval (CI): 0.03-11.69, P < 0.05). The difference in the amount of improvement was in favor of the single-level group at all follow-up times (Figure 1).

There were no differences between the groups in the amounts of improvement in SF-36v2 PCS or MCS, VAS Leg Pain, or VAS Back Pain. By 24 months, 80% (16/20) of the single-level patients and 64% (7/11) of the two-level patients had completely or mostly returned to normal activities (Table 4) (P = 0.36).

**Table 4:** Return to Activities and Patient Satisfaction by Follow-Up and Number of Levels Bold: P < 0.05 for differences between single-level and two-level instrumented PLF

		Improvement over baseline				
	Number	Six weeks	Three months	Six months	12 months	24 months
		N_1_ = 24; N_2_ = 14	N_1_ = 24; N_2_ = 15	N_1_ = 23; N_2_ = 15	N_1_ = 23; N_2_ = 15	N_1_ = 20; N_2_ = 11
Return to activities						
Single level	Completely	4 (15.4%)	4 (16.7%)	10 (43.5%)	10 (43.5%)	12 (60%)
	Mostly	8 (30.8%)	11 (45.8%)	8 (34.8%)	7 (30.4%)	4 (20%)
	Some	10 (38.5%)	4 (16.7%)	3 (13%)	5 (21.7%)	3 (15%)
	None	4 (15.4%)	5 (20.8%)	2 (8.7%)	1 (4.4%)	1 (5%)
Two levels	Completely	2 (14.3%)	2 (13.3%)	2 (13.3%)	4 (26.7%)	4 (36.4%)
	Mostly	2 (14.3%)	4 (26.7%)	9 (60%)	5 (33.3%)	3 (27.3%)
	Some	6 (42.9%)	9 (60%)	3 (20%)	5 (33.3%)	4 (36.4%)
	None	4 (28.6%)	0 (0%)	1 (6.7%)	1 (6.7%)	0 (0%)
Outcome of surgery						
Single level	Excellent	16 (64%)	16 (64%)	16 (72.7%)	17 (73.9%)	12 (60%)
	Good	5 (19.2%)	7 (28%)	3 (13.6%)	3 (13%)	4 (20%)
	Fair	3 (11.5%)	2 (8%)	3 (13.6%)	2 (8.7%)	3 (15%)
	Poor	2 (7.7%)	0 (0%)	0 (0%)	1 (4.4%)	1 (5%)
Two levels	Excellent	6 (42.9%)	3 (20%)	8 (53.3%)	8 (53.3%)	4 (36.4%)
	Good	6 (42.9%)	12 (80%)	6 (40%)	6 (40%)	3 (27.3%)
	Fair	1 (7.1%)	0 (0%)	1 (6.7%)	1 (6.7%)	4 (36.4%)
	Poor	1 (7.1%)	0 (0%)	0 (0%)	0 (0%)	0 (0%)

At the 24-month follow-up, 80% (16/20) of the single-level patients and 64% (7/11) of the two-level patients rated their surgery as being excellent or good (P = 0.36)

There was one reoperation (single level), two postoperative infections (both single level), and one dural tear (two levels). The reoperation occurred three months postoperatively and was performed to reposition a symptomatic pedicle screw that was thought to be impinging on a nerve root. One patient developed a postoperative wound seroma at 35 days post-procedure, which was treated with an incision and drainage, and the infection resolved without sequelae within two days after onset. The other patient experienced a surgical site infection at 25 days post-procedure and was rehospitalized and treated with an incision and drainage; the infection resolved without sequelae 69 days after onset. One patient experienced a headache and bilateral leg pain at three days post-procedure; this was determined to be a result of a dural tear, and the patient was treated with a dural repair. This patient’s headache and bilateral leg pain resolved without sequelae 10 days after onset.

## Discussion

In the current study, both single-level and two-level patients demonstrated high fusion rates in association with improvements in pain, functional, and QoL outcomes, as well as high satisfaction levels. Patients who underwent single-level PLF had better ODI outcomes, but similar pain and generic health-related QoL outcomes compared with patients who underwent two-level PLF. The fusion rate for single-level patients was nominally higher at 12 and 24 months, but this difference did not reach statistical significance. Return to normal activities and satisfaction with the outcome of surgery were better at three months postoperative following single-level PLFs compared with two-level PLFs.

The better ODI and patient satisfaction outcomes in single-level versus two-level procedures observed in this study could be due to more extensive surgery in the two-level patients, more possibilities for slower recovery due to multilevel pathology, or unadjusted differences in patient populations. Although not statistically significant, we found a slightly higher fusion rate in single-level patients. It is possible that the improved fusion rate contributed to superior ODI and patient satisfaction; however, the relationship between fusion status and functional outcomes or patient satisfaction is complex and beyond the scope of the current analysis.

There is no agreement in the literature concerning the minimal clinically important difference (MCID) for the ODI. The values proposed are 30% improvement [14], 10-point improvement [15], and five-point improvement [16]. The average improvements observed in our study exceeded all of these proposed MCID values in both groups and at all follow-up times. Furthermore, the degree of improvement in the single-level fusions was six points higher than the two-level PLFs, which exceeds the MCID estimate of five points.

Inage et al. compared single-level, two-level, and three-level instrumented PLFs [17]. They noted lower fusion rates for three-level PLF patients, but there were no differences in the final functional and pain outcomes among the groups. However, their statistical approach was limited as it did not compare the degree of improvement (i.e., change) but only compared the final values. A review of their summary data for the ODI showed a 27-point improvement in the single-level group, eight-point improvement in the two-level group, and 12-point improvement in the three-level group, results that are consistent with our findings.

While we found some differences in single-level versus two-level PLF, they do not necessarily impact clinical decision-making (how many levels to fuse) or any other management decisions. However, these differences can be used to advise patient expectations and prognosis.

A review of the literature indicates that reoperation rates, including procedures involving the adjacent segments, are higher for instrumented constructs than non-instrumented multilevel PLFs [9]. In our study, which included patients undergoing instrumented PLF, there was only one reoperation, which was for a symptomatic screw.

There are limitations to our study. First, we had a relatively small sample size and consequently less statistical power. However, even with this sample size, we were able to identify differences in ODI outcomes between single-level and two-level PLFs. Second, our comparison between single-level and two-level PLFs was nonrandomized. The election of levels for PLF was determined by surgeons’ judgment and based on patient pathology; therefore, it may not be possible to conduct a randomized study to better elucidate this issue. We used a prospective cohort design, which is the next best design option after a randomized controlled trial. In addition, we did not break out the outcomes of those individuals within each group who fused and consequently cannot speculate on the relationship of fusion to functional outcomes. The study was powered primarily to assess fusion, and with only a small number of patients not achieving radiographic fusion, we felt such a sub-analysis would lack statistical power. Similarly, radiographic studies were only assessed for fusion; we did not evaluate overall sagittal balance or segmental alignment. The purpose of this study, however, was to assess the outcomes of single-level and two-level PLF; a detailed analysis examining the reasonsfor the differences in outcomes is beyond the scope of the present study. Finally, the two-year follow-up rates were under 80%, which may be related to patients who experienced good outcomes choosing not to attend their scheduled postoperative follow-up visits occurring 12 months or longer after surgery [18].

## Conclusions

The results of this study suggest that both single-level and two-level PLFs are effective surgeries for select patient populations. Fusion rates trended higher for single-level PLFs compared with two-level PLFs. Single-level PLF patients appear to have better functional outcomes and report higher satisfaction compared with two-level PLF patients. While we found some differences in single-level versus two-level PLF, these differences do not necessarily impact how many levels to fuse or any other clinical management decisions. These differences, however, can be used to advise patient expectations and prognosis.
